# Lactation Support and Breastfeeding Duration in Jaundiced Infants: A Randomized Controlled Trial

**DOI:** 10.1371/journal.pone.0119624

**Published:** 2015-03-06

**Authors:** Catherine M. Pound, Katherine Moreau, Kristina Rohde, Nick Barrowman, Mary Aglipay, Ken J. Farion, Amy C. Plint

**Affiliations:** 1 Department of Pediatrics, Children’s Hospital of Eastern Ontario, Ottawa, Ontario, Canada; 2 Department of Pediatrics, University of Ottawa, Ottawa, Ontario, Canada; 3 Clinical Research Unit, Children’s Hospital of Eastern Ontario, Ottawa, Ontario, Canada; University of Alabama at Birmingham, UNITED STATES

## Abstract

**Objectives:**

Neonatal jaundice is the most common problem in full-term infants during the immediate post-natal period. We examined the effect of a lactation support intervention on breastfeeding duration in hospitalized jaundiced infants.

**Study Design:**

We conducted a randomized controlled trial with a qualitative component involving mothers of hospitalized jaundiced breastfed infants <4 weeks of age. Mothers receiving the intervention met with an International Board-Certified Lactation Consultant in hospital and 1–3 times post discharge. Both groups received the standard care for jaundice. The primary outcome was exclusive breastfeeding at 3 months. To the exception of research assistants enrolling participants and completing qualitative interviews, all research staff, investigators and statisticians were blinded to group assignment. Qualitative interviews elicited feedback on breastfeeding experiences for both groups.

**Results:**

99 participants were recruited, and 86 analyzed for primary outcome. There was no difference in exclusive breastfeeding at 3 months between groups (RR 0.84, 95% CI 0.56–1.24, p = 0.40) or in the secondary outcomes. 31 participants were included in the qualitative analysis. Participants in the intervention group described an increase in comfort and confidence levels with breastfeeding. Participants in the control group reported limited lactation support.

**Conclusions:**

Our hospital-based lactation support program did not result in a higher proportion of mothers exclusively breastfeeding at 3 months compared to current hospital standard care. Qualitative feedback from the intervention group suggests that mothers’ confidence was increased, which is linked to breastfeeding duration. The decision to breastfeed is multifactorial and hospital-based lactation support may be only a small piece of the puzzle in hospitalized jaundiced infants. Further studies may be needed to fully elucidate the impact of an in-hospital lactation support program on successful breastfeeding for these infants.

**Trial Registration:**

ClinicalTrials.gov NCT00966719 https://www.clinicaltrials.gov/ct2/show/NCT00966719?term=Lactation+Support+and+Breastfeeding+Duration+in+Jaundiced+Infants%3A+a+Randomized+Controlled+Trial&rank=1

## Introduction

Breastfeeding confers many advantages to infants, mothers, families, and society [1] and is the normal nutrition for the newborn infant. Human milk infant feeding decreases the incidence of infectious diseases [2–4] and enhances the immunologic status of the newborn [1]. Exclusive breastfeeding is therefore recommended for the first six months of life [1,5,6].

Neonatal jaundice is the most common problem in full-term infants during the immediate post-natal period [7]. Mothers of infants admitted to hospital with jaundice experience guilt [8], and feelings of failure and inadequacy [9]. Maternal confidence is known to be a strong predictor of breastfeeding duration, with lack of confidence in breastfeeding skills leading to a higher likelihood of weaning in the first six weeks post-partum [10]. Research suggests that mothers of infants admitted to the hospital with a diagnosis of jaundice have a higher rate of breastfeeding discontinuation than infants in the general population [11], presumably partly due to a lack of lactation support while in the hospital. Previous studies show that lactation support, such as educational programs, can significantly improve rates of breastfeeding at 2 to 6 months of age [12,13], with absolute increases of up to 37% in exclusive breastfeeding at 3 months with breastfeeding promotion interventions [13].

The clear health benefits provided by breastfeeding, and the concern of early breastfeeding discontinuation in hospitalized jaundice infants, led us to investigate the effect of a lactation support intervention on breastfeeding duration in infants admitted to the hospital with jaundice. We hypothesized that mothers who received a formal hospital-based lactation support intervention would be more likely to exclusively breastfeed their infants at 3 months than mothers who received the current standard of care.

## Methods

The protocol for this trial and supporting CONSORT checklist are available as supporting information; see S1 Protocol and S1 CONSORT Checklist.

### Study Design

We conducted a randomized controlled trial with a secondary qualitative component. Blinding of study participants was not feasible due to the nature of the intervention. With the exception of the research assistants responsible for recruiting mothers, completing the qualitative follow-up interviews and their subsequent analyses, all research staff, including the investigators and statisticians, were blinded to group assignment. The Children’s Hospital of Eastern Ontario (CHEO) Research Ethics Board approved this study. The trial was registered on www.clinicaltrials.gov, identifier number NCT 00966719.

### Study Setting and Participants

Mothers of all infants admitted to CHEO with jaundice during the study period were screened for eligibility. CHEO, located in Ottawa, Canada, is a tertiary-care pediatric hospital. Mothers of infants ≤4 weeks of age admitted to hospital with jaundice and breastfeeding any amount were eligible. Mothers were deemed ineligible to participate if their infants were: (a) exclusively formula-fed, (b) admitted with predominantly conjugated hyperbilirubinemia, (c) admitted with anatomical abnormalities that would interfere with breastfeeding, (d) neurologically impaired, (e) admitted to the neonatal intensive care unit directly after birth, (f) fed via enteral tubes, or (g) the result of a multiple birth. In addition, mothers who had previous breast surgery, did not understand English or French, or were foster or adoptive mothers to the admitted infant were also ineligible. We planned to recruit from October 2009 to April 2011, but continued until October 2012 due to slow recruitment.

### Study Protocol

A computer-generated randomization schedule was prepared in advance for the initial recruitment period, and before every extension period, by a statistician with no role in recruitment. The sequence was composed of randomly permuted blocks of 4 or 6. Group assignments were concealed in sequentially numbered opaque envelopes kept in a secure location. A research assistant regularly reviewed the list of admissions to identify all infants admitted to the hospital with jaundice. The research assistant completed eligibility screening, obtained written, informed consent, and allocated mothers to their study group.

#### Intervention Group

Mothers randomized to the intervention group received the current standard of medical care for jaundice at CHEO (i.e. phototherapy and intravenous fluids) and met with one of two International Board of Lactation Consultant Examiners-certified lactation consultants (LC) once during the infant’s hospitalization. The LC’s intervention was based on established clinical practice guidelines [14] and included a review of the benefits of breastfeeding as well as an assessment of the mother’s breastfeeding techniques, with correction as needed. Mothers were taught how and when to use a breast pump and how to store breast milk. Breast pumps and related equipment were provided at no cost to participating mothers for up to 6 weeks.

Once the infant was discharged, the LC offered three weekly half-hour follow-up sessions at CHEO. We initially requested that mothers attend all three sessions but later changed the requirement to a minimum of one session, as many mothers refused to participate in the study due to the number of required follow-up visits. Sessions were available during day and evening hours. Breastfeeding techniques were reviewed and corrected as necessary, and the mother’s questions or concerns were addressed. Once the follow-up sessions were over, the LC provided the mother with a list of resources should further breastfeeding issues arise.

The two LCs both had over 10 years of experience. A computer randomly generated the LC’s call schedule. Each LC followed up with the patients she initially saw, unless unforeseen circumstances such as illness occurred, necessitating follow-up by the other consultant. We further ensured standardization of the intervention by having the LCs generate a formal tool to assess the mother and infant.

At the end of the study period, an International Board Certified Lactation Consultant, independent of the study, was asked to review 10% of the files kept by the study LCs to ensure that advice provided to the mothers met the standard of care, as defined by the International Board of Lactation Consultants.

#### Control Group

Mothers randomized to the control group received the current CHEO standard of medical care for jaundice (fluids and phototherapy). They received no formal, standardized breastfeeding support. As part of the standard of care at our institution, mothers in this group could receive advice and recommendations from the nurses or physicians caring for the infant while in hospital, but such advice or recommendations was not standardized. No care was withheld from the control group, as LCs are not available at our institution. However, mothers in both groups could consult private LCs as well as public health nurses once discharged from the hospital.

### Measurements

Baseline information about the mothers (e.g., demographics, maternal age, number of children, and previous breastfeeding experience) and their infants (e.g., gestational age, birth weight, admission weight) was recorded at the time of study enrollment. Follow-up measurements were collected until the infant was 6 months old. Data on breastfeeding duration was collected at various point intervals directly from the mothers, as maternal recall has been shown to be valid and reliable for up to 3 years [15]. Mothers were given a diary upon discharge from the hospital, and were instructed to fill out the diary in order to record information on formula and solid food introduction; timing of return to work; number of daily formula feeds and sessions at the breast at different points in time; any hospitalizations and reason; visits to primary care physicians and reasons; discussion with primary care physicians regarding breastfeeding; and visits to lactation support services.

A research assistant, blinded to group allocation, phoned participants 1 week after hospital discharge to remind them to complete their study diaries. Mothers were reminded by telephone to complete their diaries when the infant was 2, 3, 4 and 6 months old. We initially planned to collect feeding information both via diary and follow-up phone calls, so as to cross-reference the mothers’ answers for accuracy. However, some mothers did not return the diaries and others could not be reached via phone. Thus, 16 months into the study, we collected data by telephone for only those mothers who did not return the diaries.

To obtain feedback on the lactation support intervention on breastfeeding, we also included a secondary qualitative data collection component. This qualitative component was conducted with a subgroup of mothers from both the intervention and control groups.

For the intervention group, the interview guide featured 10 open-ended questions that focused on the mothers’ perceptions of the intervention. Interviews occurred 1 week after the final lactation consultant session. Questions focused on mothers’ perceptions of breastfeeding prior to the intervention, their expectations of the study lactation consultant, and their experience in the intervention.

Conversely, for the control group, the interview guide featured 7 open-ended questions that focused on the mothers’ perspectives on the standard of care they received for lactation support while in the hospital. Again, interviews occurred 1 week after hospital discharge

### Outcomes

The primary outcome measured was exclusive breastfeeding when the infant reached 3 months of age, or 3 months corrected, if the infant was born prematurely. Exclusive breastfeeding was defined as no milk intake other than breast milk. Secondary outcomes included partial breastfeeding at 3 and 6 months, exclusive breastfeeding at six months, number of re-hospitalizations and physician visits in the first 6 months of life, and amount and type of lactation support given by child’s primary physician in first 6 months of life. We also a priori planned to compare exclusive breastfeeding proportions in our control group with those described in the Ottawa population [16].

### Sample size

We assumed 50% as the baseline rate of exclusive breastfeeding at 3 months, based on the City of Ottawa’s breastfeeding rates [16] and results of our previous study [11]. We powered our study to detect an absolute 25% increase in the rate of breastfeeding at 3 months. Such an increase was considered plausible based on a meta-analysis of randomized controlled trials combining educational and support programs that demonstrated increased breastfeeding rates of 36% (95% CI 22–49%) [12]. Setting the probability of type I error at 0.05, a sample size of 58 mothers per group would result in a power of 80% to detect a 25% increase in breastfeeding rates. Given that previous studies of young infants conducted at our institution had shown high follow-up rates when primary outcomes were determined through phone follow-up [17,18], we estimated our loss to follow-up to no more than 7.5%. To account for this loss, we needed to recruit 62 patients in each group. For the qualitative component of the study we aimed to recruit 20 randomly selected mothers from each group. Sample size was calculated using PS Software (version 3) [19]

### Quantitative Analysis

The primary analysis followed the intention-to-treat principle and involved all patients with a recorded primary outcome. The primary analysis was a comparison between groups of exclusive breastfeeding proportions at 3 months using Fisher's exact test. Risk ratios and 95% confidence intervals were also computed. All tests were two-sided, with p-values less than 0.05 considered statistically significant. Time to discontinuation of exclusive breastfeeding was compared between groups graphically using Kaplan-Meier curves, and statistically using the log-rank test. Breastfeeding proportions were compared to those in the Ottawa community as determined by the Infant Care Survey [16] using a one-sample binomial test. Other secondary outcomes were tested using Fisher’s exact test and Poisson regression models where appropriate. Imputation of data missing due to loss to follow-up was not performed because it would require strong assumptions, which may be hard to justify [20]. SPSS version 22 was used to perform all analyses.

### Qualitative Analysis

A conventional qualitative content analysis [21] was used to analyze the interview data. This approach ensured that the coding scheme was derived inductively and flowed directly from the data. A trained qualitative research assistant independently read the transcripts multiple times to obtain a sense of the whole and developed a coding scheme to analyze the data [22]. To ensure the trustworthiness of the analysis, the research assistant engaged in a peer debriefing process with another qualitative analyst. To enhance the credibility of the findings, each participating mother was also provided with the opportunity to verify her interview transcript.

## Results

### Patient recruitment and baseline characteristics

**Fig 1:**
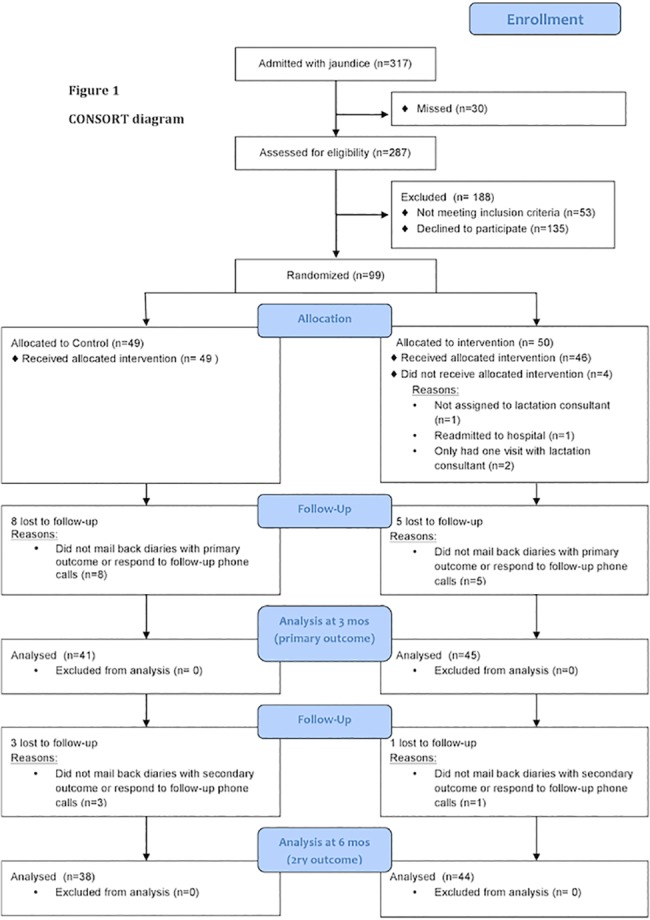
CONSORT diagram.

Fig. 1 and Table 1 outline recruitment and study participant characteristics. During the study period, 317 infants with jaundice were admitted to hospital; 234 mothers were eligible and 99 were enrolled with 50 allocated to lactation support and 49 to standard of care. Primary outcome data was available for 45 women in the intervention group and 41 in the control group. There were 4 protocol deviations among patients in whom primary outcome data was available; one participant allocated to the intervention group did not participate in any lactation consultant sessions and three participants only received the in-hospital lactation consultant session.

**Table 1:** Demographics and clinical characteristics of all participating mothers.

	Control (n = 49) n (%)	Intervention (n = 50) n (%)
Language		
English	43 (87.8)	50 (100.0)
French	6 (12.2)	0 (0.0)
Age		
15–20	0 (0.0)	2 (4.0)
21–25	9 (18.4)	6 (12.0)
26–30	13 (26.5)	11 (22.0)
31–35	18 (36.7)	23 (46.0)
36–40	7 (14.3)	6 (12.0)
>40	2 (4.1)	2 (4.0)
Highest level of education		
Completed high school	4 (8.2)	4 (8.0)
Vocational/ technical training (post high school)	13 (26.5)	13 (26.0)
Some university training	4 (8.2)	5 (10.0)
Completed university	28 (57.1)	28 (56.0)
Combined household income^a^		
Under $30 000	7 (14.6)	4 (8.2)
$30 000- $69 000	9 (18.8)	8 (16.3)
$70 000- $100 000	12 (25.0)	10 (20.4)
Above $100 000	18 (37.5)	21 (42.9)
Declined to answer	2 (4.2)	6 (12.2)
Mother smokes at home		
Yes	0 (0.0)	1 (2.0)
Marital status^b^		
Married/ common-law	47 (97.9)	45 (90.0)
Single	1 (2.1)	5 (10.0)
Number of prior children		
none	31 (63.3)	22 (44.0)
1	14 (28.6)	22 (44.0)
>1	8(8.1)	6 (12.0)
Number of prior breastfed children		
0	31 (63.3)	24 (48.0)
1	15 (30.6)	23 (46.0)
>1	3 (6.1)	3 (6.0)
Received BF support with prior infants^c^		
Yes	15 (86.3)	14 (51.9)
Infant received formula prior to hospitalization		
Yes	22 (45.8)	27 (54.0)
Received medical care from:		
Obstetrician	29 (59.2)	32 (64.0)
Family physician	21 (42.9)	29 (58.0)
Midwife	15 (30.6)	8 (16.0)
No prenatal care	0 (0.0)	3
Attended prenatal classes		
Yes	21 (42.9)	17 (34.0)
Planning to return to work before child is 1 year of age		
Yes	12 (25.0)	10 (20.0)
Mean age at admission to CHEO in days (mean, SD)	5.9 (4.0)	6.4 (4.0)
Baby’s birth weight (kg)	3.3 (0.5)	3.3 (0.6)
Mean gestational age at birth (mean, SD)	38.3 (1.5)	38.3 (1.5)

^a^n = 97,

^b^n = 98,

^c^n = 45

Study subject recruitment was much more difficult than anticipated and we were unable to meet our target sample size of 62 patients per group despite extending our recruitment period by 18 months over our initial time estimate. During the design of our study, all infants with jaundice within Ottawa were admitted only to our hospital (CHEO). Shortly into our recruitment period, community hospitals began readmitting infants born at their hospital if they required treatment for jaundice. This change in admission practices dramatically reduced the number of infants with jaundice admitted to our centre. Community-based birth hospitals, as centres that provide labour and delivery care, routinely provided lactation consultant services and thus we were unable to conduct the study at these sites. Moreover, recruitment was also difficult because, while most mothers were very willing to meet with the lactation consultant in hospital, many reported that they would not be returning for the follow-up sessions due to commitments to other children, transportation difficulties, or fatigue and thus did not wish to enrol in the study.

### Breastfeeding outcomes

**Fig 2:**
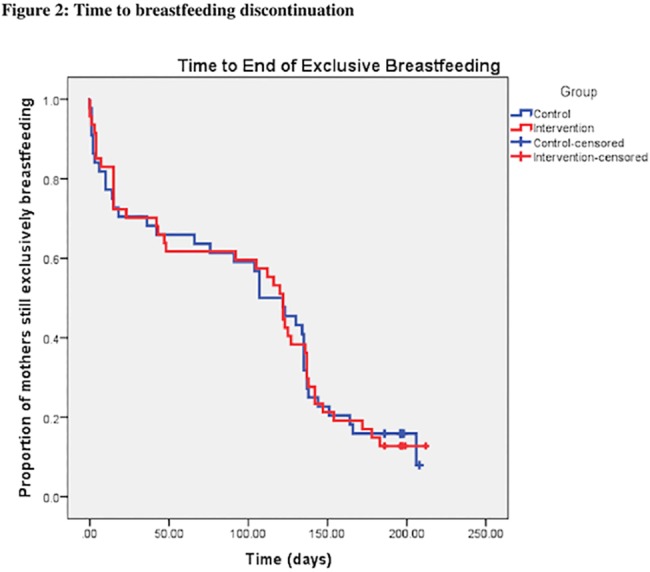
Time to breastfeeding discontinuation. Control, Intervention, Control-censored, Intervention-censored.

There was no significant difference between groups in exclusive breastfeeding at 3 months (Table 2). The groups had similar partial breastfeeding proportions at 3 and 6 months and exclusive breastfeeding proportions at 6 months. The number of mothers seeking breastfeeding help did not differ between both groups at 3 and 6 months. There was also no statistically significant difference between the intervention and control groups with respect to time to discontinuation of breastfeeding (p = 0.97). The median time to end of breastfeeding was 107 days (95% CI: 78.8–135.2) for the control and 122 days (95% CI: 78.6–119.74) for the intervention group (Fig. 2).

**Table 2:** Breastfeeding outcomes and subsequent health care utilization.

Risk estimates^a^	Control group: n (%)	Intervention group: n (%)	Risk ratio^b^		95% confidence interval	*P* value
**Primary Outcome**						
Exclusive breastfeeding at 3 months	24/41 (58.5)	22/45 (48.9)	0.84		0.56–1.24	0.40
**Secondary Outcomes**						
Partial breastfeeding at 3 months	39/41 (95.1)	43/45 (95.6)		1.00	0.91–1.10	1.00
Exclusive breastfeeding at 6 months	6/38 (15.8)	8/44 (18.2)		1.15	0.44–3.02	1.00
Partial breastfeeding at 6 months	31/38 (81.6)	37/44 (84.1)		1.03	0.85–1.26	0.78
Number of mothers seeking breastfeeding help at 3 months	7/41 (17.1)	7/45 (15.6)		0.91	0.35–2.38	1.00
Number of mothers seeking breastfeeding help at 6 months	7/38 (18.4)	11/44 (25.0)		1.36	0.58–3.15	0.60
Number of infants with any subsequent hospital admissions for jaundice	1 (2.0)	3 (6.0)		2.94	(0.32–27.30)	0.32
Number of infants with any hospital admissions unrelated to jaundice	3 (6.1)	2 (4.0)		1.50	0.95–2.38	0.09
Number of infant-physician encounters per year (median, IQR)	9.9 (6.3–12.0)	9.2 (6.0–12.0)		1.03	0.83–1.27	0.77

^a^comparing intervention relative to control

^b^ Poisson regression analyses were used to compute incidence rate ratios for number of infants with any hospital admissions unrelated to jaundice and number of infant-physician encounters per year through. All other outcomes were tested using Fisher’s exact test.

There was no significant difference in the number of re-hospitalization for jaundice and non-jaundice related causes, as well as the number of physician encounters in the first 6 months of life between groups (Table 2).

Partial breastfeeding proportions at 3 and 6 months were also compared between our control group and the proportions reported in the Infant Care Survey [16] and were found to be much higher in our study population (Table 3).

**Table 3:** Comparison of proportions of mothers breastfeeding in study control group and community in general.

	Study control group (%)	Infant Care Survey group^a^ (%)	*P* value^b^
Exclusive breastfeeding at 3 months	59	50	0.698
Partial breastfeeding at 3 months	95	71	<0.0001
Exclusive breastfeeding at 6 months	16	39	0.004
Partial breastfeeding at 6 months	82	60	0.007

^a^ Ottawa Public Health. Infant Care Survey 2005 [16]

^b^P values obtained through one-sample binomial tests

Possible predictors of missing outcome at 3 months were investigated including language, age, education, income, and marital status. Non-respondents were found to be significantly less likely to be married (p = 0.03).

There were no adverse events reported in the intervention group; no child was noted to have lost weight, become more jaundiced, have a decreased arousal level, or have any other symptom of concern to the lactation consultant at the follow-up visits.

### Qualitative interviews and results

Thirty-one mothers participated in the qualitative interviews (13 from the control and 18 from the intervention group).

#### Intervention group

In the intervention group, mothers perceived the LCs, as well as the breast pumps and follow-up appointments, to be very helpful. Participants reflected on how the LCs increased their comfort and confidence levels with breastfeeding; enhanced their sense of encouragement and reassurance; motivated them to continue breastfeeding; strengthened their emotional wellbeing; and empowered them with the knowledge and skills to pursue breastfeeding with their subsequent children. For instance, one mother stated:

*I felt more confident because I knew he was actually getting food*. *I guess I felt more knowledgeable*. *I knew different things and ways to do them*. *It was not that the way I was doing it before was necessarily not a good way because it might be better later on*, *but the way she taught me now is much better for his size and the way he eats fast*. *It was very beneficial*.

Many mothers also appreciated the friendly and comforting demeanour, as well as the expertise of the LCs. Several participants recognized the specialized support and training of the LCs, clearly distinguishing them from other health care professionals in the hospital:

*I was happy to meet with her*, *just having the extra expertise and additional training that she might have*. *We were welcomed to learn about it and she was very patient and understanding*. *That was nice considering the circumstances of being rather stressed with the baby in the hospital*.

Moreover, in regards to the breast pumps and follow-up sessions, multiple mothers thought the loaning of the breast pump was beneficial. For example, one participant stated, “*the pump was something that enabled me to keep up my milk supply*. *It was very helpful*.”

Although some mothers felt the follow-up sessions were inconvenient, many appreciated them. They recounted how they could ask questions and weigh their babies, which enabled them to track their infant’s growth. A participant nicely described this perspective about the follow-up sessions by stating, “*even more useful than in the hospital because in the hospital you are just leaving the hospital and feeling nervous or whatever*. *You do not have all the questions you might want*. *The follow-up sessions [are] very valuable in going through each week*.”

#### Control group

In the control group, mothers reflected on the adequacy of lactation support while admitted to hospital. Feedback on the support offered in hospital was negative. Participants described a lack of support and knowledge from the healthcare providers. Most participants also expressed uncertainty in terms of their breastfeeding practices. The following quotations illustrate this reservation:

*We were just not sure if we were feeding enough or not*, *but that was not a question the nurses could answer because they could only tell by the poop—that is what I heard*. *After that I stopped asking questions*.

*If I could have had somebody like that to advocate for me a little bit on the breastfeeding side*, *if the doctors or even the nursing staff could have a little more knowledge about that and understanding*, *even just for the mother’s case*, *that sitting there without any way to feed or even pump is just not good for your milk supply*.

## Discussion

In this randomized controlled trial that compared a structured lactation consultant support program to the standard of care for hospitalized jaundiced newborns, we did not find any differences in exclusive or partial breastfeeding proportions up to 6 months. This result is surprising given the published literature. A recent Cochrane review found that both professional and lay support were effective in prolonging breastfeeding, with professional support being the most effective of the two [23]. In a different systematic review, when focusing on randomized controlled trials combining lactation support with educational programs in developed countries, combined education and support strategies increased short-term breastfeeding rates by 36% [12]. Finally, in the PROBIT trial [13], a randomized controlled trial evaluating the effectiveness of a breastfeeding promotion intervention in the Republic of Belarus, an absolute increase of almost 37% in the prevalence of exclusive breastfeeding at 3 months was found in the intervention group. It is worth noting the sharp drop in breastfeeding proportions in both participant groups in the first month as well as between 3 and 4 months (Fig. 2). These time points may well represent crucial times that breastfeeding interventions should focus on.

Despite there being no difference in overall breastfeeding outcomes in our study, the secondary qualitative findings were very positive towards our lactation support intervention. Participants described an enhanced sense of encouragement and reassurance, increased motivation and empowerment to continue breastfeeding, and an enhanced sense of emotional wellbeing. Mothers in the intervention group felt overall more confident with breastfeeding in comparison to those in the control group. This is crucial as maternal confidence is a strong predictor of breastfeeding outcome [10], with lack of confidence leading to a higher likelihood of weaning in the first 6 weeks post-partum [23]. In the control group, most participants recognized that the support provided by nurses and physicians was limited. This is consistent with other studies showing that physicians and residents lack the skills to offer proper guidance to lactating mothers [24–31].

Our study has some limitations. Recruitment was much more difficult than anticipated, and we were not able to reach our target sample size of 62 mothers per group. Given the very large confidence interval around our risk ratio (0.56 to 1.24) for exclusive breastfeeding at 3 months, it is possible that the intervention could result in an improvement in breastfeeding in this population. It would appear however that the effect is likely small, and would require a much larger sample size. Thus, even if we had reached our target sample size, we likely would not have seen an effect of the intervention. The effect of intervention may have been attenuated since it appears that women with a strong desire to breastfeed were more likely to enrol in the study (the breastfeeding proportions in our control group were found to be much higher than those in the general population). We also noted that more mothers in the control group compared to the intervention group reported receiving breastfeeding support with previous infants and this may also have increased the proportion of mothers that maintained breastfeeding in the control group.

## Conclusions

In conclusion, given the positive health outcomes that breastfeeding confers to infants and mothers, and the large economic benefits to society gained from a decrease in disease burden, continued attention should be dedicated to the support of breastfeeding mothers of hospitalized infants. While we did not find a difference in breastfeeding proportions between groups, the qualitative findings clearly illustrated the benefits of our intervention to mothers. The decision to breastfeed is multifactorial and hospital-based lactation support may be only a small piece of the puzzle in hospitalized jaundiced infants. Further studies may be needed to fully elucidate the impact of an in-hospital lactation support program on successful breastfeeding for these infants.

## Supporting Information

S1 Protocol (DOC)

S1 CONSORT Checklist (DOC)
