# Supplementary material for: Lactation Support and Breastfeeding Duration in Jaundiced Infants: A Randomized Controlled Trial
Source: PLoS One. 2015 Mar 6;10(3):e0119624. doi: 10.1371/journal.pone.0119624 (PMC4351896; doi:10.1371/journal.pone.0119624)
Supplement: S1 Protocol — (DOC) [file pone.0119624.s001.doc]

**The Impact of a Breastfeeding Support Intervention on Breastfeeding Duration in Jaundiced Infants Admitted to a Tertiary Care Centre Hospital; a Randomized Controlled Trial**

**Principal Investigator**

Catherine M. Pound, MD

Assistant Professor, Department of Pediatrics, University of Ottawa

Children's Hospital of Eastern Ontario

Department of Pediatrics

401 Smyth Road

Ottawa, Ontario

K1H 8L1

Tel: 613-737-7600 x 2571

Fax: 613-738-4878

[cpound@cheo.on.ca](mailto:cpound@cheo.on.ca)

**Co-investigators:**Amy C Plint, MD, MSc

Associate Professor, Departments of Pediatrics and Emergency Medicine, University of Ottawa

Senior Investigator, Children’s Hospital of Eastern Ontario Research Institute

Director of Research, Division of Emergency Medicine, Children’s Hospital of Eastern Ontario

Katherine Moreau, PhD(c)

Program Evaluator/Research Coordinator

Clinical Research Unit, Children’s Hospital of Eastern Ontario Research Institute

Ken Farion, MD

Associate Professor, Departments of Pediatrics and Emergency Medicine, University of Ottawa

Associate Medical Director and Director of Informatics, Division of Emergency Medicine, Children’s Hospital of Eastern Ontario

Nick Barrowman, PhD

Senior Statistician and Investigator, Clinical Research Unit, Children’ Hospital of Eastern Ontario Research Institute

Assistant Professor, Department of Pediatrics, University of Ottawa

Adjunct Research Professor, School of Mathematics and Statistics, Carleton University

Rhonda Correll, BScN

Operations Director, Clinical Research Unit, Children’s Hospital of Eastern Ontario Research Institute

**Brief Description of Project**

Breastfeeding decreases the risk of infectious illnesses in infants, strengthens the mother-infant bond and decreases health care costs by making infants healthier. There is evidence that when infants with jaundice are hospitalized their mothers are at risk of stopping breastfeeding.

This study’s main objective is to determine the effect of a breastfeeding intervention on breastfeeding duration in jaundiced infants. Mothers of infants in the intervention group will meet with a lactation consultant on four occasions. Mothers of infants in the control group will receive regular hospital care. Follow-up of mothers will continue until the infant is 6 months old. Information will be collected on length of time that infants are fed only breast milk, future visits to health care providers, mothers’ perception of their physicians’ attitudes towards breastfeeding, and mothers’ experiences at the hospital, as well as feedback on the intervention.

The results of this study will clarify the importance of offering breastfeeding support to mothers of these infants and help determine whether there is a need for lactation specialists in children’s hospitals. It will allow us to determine the impact of such intervention on children’s health in their first 6 months of life, as well as collect information on support offered to breastfeeding women by primary care physicians.

**The Impact of a Breastfeeding Support Intervention on Breastfeeding Duration in Jaundiced Infants Admitted to a Tertiary Care Centre: a Randomized Controlled Trial.**

**1. RESEARCH QUESTION AND OBJECTIVES**

**1.1 Overview of the Research Problem:**

A study is proposed that will assess the effectiveness of a breastfeeding support intervention for infants admitted to the hospital with jaundice. This research will evaluate the effect of this intervention on duration of breastfeeding and health care utilization.

**1.2 Study Objectives:**

*Primary Objectives*: To determine the effect of a breastfeeding support intervention on breastfeeding duration in breastfeeding infants admitted to the hospital with jaundice.

*Secondary Objectives:*

- To determine the rate of breastfeeding failure, defined as breastfeeding cessation before the infant turns 6 months, in mothers of jaundiced infants randomized to the control group, as compared to the rate of breastfeeding failure in the general population.
- To compare subsequent healthcare utilization between groups during their first six months of life, as determined by the number of re-hospitalizations for jaundice, hospitalizations for non-jaundice related causes, as well as physician encounters.
- To determine the number of mothers seeking breastfeeding help once discharged from the hospital and compare it between both groups.
- To determine the kind and perceived effectiveness of breastfeeding support and advice given by the infant’s primary physician throughout the child’s first six months of life.

*Hypothesis*: We hypothesize that, among mothers of breastfeeding infants who are admitted to hospital with jaundice, mothers who receive formal breastfeeding support will be more likely to exclusively breastfeed their infant at 3 months than mothers who receive the current standard of care.

**2. BACKGROUND/PRESENT STATE OF KNOWLEDGE**

**2.1 Summary of the problem:**

Breastfeeding confers many advantages to infants, mothers, families, and society in general^1^. There is strong evidence that human milk feeding decreases the incidence of many infectious diseases^2^ and enhances the immunologic status of the newborn^1^. It has been associated with enhanced performance on neurocognitive development tests^3-4^, and has also been shown to provide important health benefits to the mother, including a decrease in risk of breast^5,6^ and ovarian cancers^7^. Breastfeeding strengthens the bond between mother and infant, delays return of ovulation and provides economic savings^1^. Given the clear advantage that breastfeeding confers to young babies, it should be strongly encouraged for the first six months of life^1^, and then continued for up to 2 years and beyond, while supplementing with appropriate complementary foods, as recommended in the World Health Organization’s *Innocenti Declaration* ^8^.

Neonatal jaundice is the most common problem in full-term infants during the immediate post-natal period^9^. There is controversy as to whether breastfeeding increases the incidence of jaundice in the first days of life. Many reports have reported an association between breastfeeding and significant hyperbilirubinemia^10-12^, but others have not substantiated this observation^13,14^. In a study conducted in Italy, neonatal jaundice was not associated with breastfeeding per se, but rather with increased weight loss after birth subsequent to fasting^9^, which can be seen with inadequate lactation.

When an infant is hospitalized, previously established patterns of breastfeeding are difficult to maintain^15^. There is evidence to suggest that mothers of young infants admitted to hospital with hyperbilirubinemia commonly experience guilt as they feel breastfeeding caused the jaundice^16^. Many of these women struggle with feelings of failure or inadequacy^15^. A study recently conducted by the principal investigator of this current study (CP) suggested that infants admitted to the hospital with hyperbilirubinemia may have a higher rate of breastfeeding discontinuation than babies in the general population^17^.

Previous studies have shown that breastfeeding support offered to various groups of mother-infant pairs significantly increased rates of breastfeeding at 2 to 6 months of age^18,19^.

Given the clear health, social and economic advantages that breastfeeding confers to mothers, infants, and society in general, there is a need to assess the effectiveness of a breastfeeding support intervention for infants admitted to the hospital with jaundice.

**2.2 Benefits of breastfeeding**

Breastfeeding is preferred for all infants, and exclusive breastfeeding of infants is recommended for the first 6 months after birth^1,20-22^. The benefits of breastfeeding to both infant and mother are well known and include superior nutritional content of human breast milk, enhanced immunologic status of the newborn, strengthening of the infant-mother dyad, delayed return of ovulation, and economic savings^1^.

Research has shown that human milk feeding decreases the incidence of multiple illnesses including bacterial meningitis^23,24^, bacteremia^24,25^, diarrhea^26-29^, respiratory tract infection^29-31^, otitis media^26,32,33^, and urinary tract infections^34,35^. A recent study conducted in Spain^36^ showed that exclusive breastfeeding lowered the risk of hospitalization as a result of infectious diseases during the first year of life. In that study, each additional month of exclusive breastfeeding would have avoided 30% of hospital admissions. A meta-analysis of 33 studies examining healthy infants in developed nations showed similar results, with formula-fed infants experiencing a tripling in severe respiratory illnesses compared with those who were exclusively breastfed for 4 months^30^. Based on these results, breastfeeding promotion can lead to important economical gains from a societal perspective.

According to the most recent Ottawa Public Health Survey^37^, breastfeeding rates in the general population are as follows: 91% of women initiate breastfeeding, with 56% practicing exclusive breastfeeding (no fluids other than breast milk). At 3 months, 71% of women continue to breastfeed, with 50% doing exclusive breastfeeding. This number drops to 60% for any breastfeeding at 6 months, with 39% of women exclusively breastfeeding.

**2.3 Breastfeeding in hospitalized children**

Published reports^15^ have shown that previously established patterns of breastfeeding are hard to maintain once an infant is hospitalized. Moreover, as mentioned, evidence suggests that mothers of infants admitted to the hospital with jaundice experience guilt^16^, as well feelings of failure and inadequacy^15^ as they often feel responsible for the child's admission to hospital. Maternal confidence has been shown to be a strong predictor of breastfeeding duration^38,39^, with lack of confidence in breastfeeding skills leading to a higher likelihood of weaning in the first six weeks post-partum^39^. There is only one study in the literature examining the rates of breastfeeding in the jaundiced population^17^, which was done by the PI of this current study. Surveys were sent to all mothers of eligible infants admitted with jaundice to the Children's Hospital of Eastern Ontario (CHEO) over a 2.5 year period. One hundred and twenty-eight (64%) out of the 200 eligible mothers returned a completed survey. Of those, 92% had a post-secondary education. Information was collected, retrospectively, on length of breastfeeding after discharge from the hospital. Length of breastfeeding for hospitalized infants was compared to that of the Ottawa population, as reported by the City of Ottawa Public Health survey^37^ in 2005. Although numbers were the same between the jaundiced group and the City of Ottawa group, it is likely that the high education level of the study participants falsely elevated the rate of breastfeeding in the jaundiced infants. As mentioned, 92% of study participants had completed a post-secondary education, as compared to 53% of women involved in the population-based survey. Given that previous reports have shown that women with lower levels of education are more likely to discontinue breastfeeding early^38^, it is reasonable to postulate that for comparable levels of education, mothers of infants admitted to the hospital with jaundice discontinue breastfeeding earlier than women in the general population.

**2.4 Breastfeeding support interventions**

There are very few studies quantifying the effects of breastfeeding promotion interventions. A recent Cochrane review found that both professional and lay support were effective in prolonging breastfeeding. More specifically, this review found that professional support had more of an impact than lay support on both partial and exclusive breastfeeding and lay support affected exclusive breastfeeding^40^. A review of four randomized controlled trials combining breastfeeding support with educational programs in developed countries found that combined education and support strategies increased short-term breastfeeding rates by 36%^18^. Short-term breastfeeding in these studies was described as anywhere between 2 to 6 months. The education interventions were primarily conducted by lactation specialists or nurses as antepartum sessions, while the support interventions varied between telephone or in-person conversations, hospital or home visits by lactation consultants, nurses or peer counselors, and combined prearranged appointments and unscheduled visits or telephone calls for problems^18^. Breastfeeding education and support had the biggest effect on initiation and maintenance of breastfeeding. Written material was not found to be beneficial to breastfeeding duration. Many of the studies however lacked scientific rigor. Most studies did not make a difference between exclusive and partial breastfeeding. Details regarding description and length of interventions as well as training of the individual delivering the educational session were often lacking. Group randomization was not always described, and in some studies, financial and gift incentives were given to the intervention groups, introducing a potential bias to the results.

A recent randomized controlled trial evaluating the effectiveness of a breastfeeding promotion intervention in the Republic of Belarus^19^ found an absolute increase of almost 37% in the prevalence of exclusive breastfeeding at 3 months in the intervention group. The chief obstetrician from each intervention maternity hospital, and the chief pediatrician from each intervention polyclinic, received the same 18-hour courses emphasizing methods to maintain lactation, promote exclusive and prolonged breastfeeding, and resolve common problems. All midwives, nurses and physicians providing care to study mothers and infants during labor, delivery, and the postpartum hospital stay, as well as all pediatricians and nurses working in the polyclinics were trained over a period of 12 to 16 months. Such a program offered the advantage of providing reliable and consistent support, with the goal of being widely available. This study was published after the Cochrane review and thus is not included in the review.

Although no study published to date has determined which aspect of a breastfeeding support intervention yields positive results, it is likely to result from a combination of factors. Breastfeeding support results in increased confidence, and maternal confidence is known to be a strong predictor of breastfeeding outcome^38,39^, with lack of confidence in breastfeeding skills leading to a higher likelihood of weaning in the first six weeks post-partum^39^.

**2.5 Availability of breastfeeding support**

In the recent CHEO study^17^, 57% of the participating mothers needed to seek breastfeeding support after discharge from the hospital, suggesting they did not receive adequate help while hospitalized. Half of these women received help from private lactation consultants. These lactation consultants have variable training and may or may not hold recognized certifications or credentials. Their fees also vary from 10$ to 90$ an hour, and therefore may not be a suitable alternative for families with financial difficulties. This indicates the need for a standardized breastfeeding support intervention that will be accessible to all.

**2.6 Breastfeeding and health care professionals**

Many studies have shown that physicians and physicians in training lack the necessary skills to offer proper guidance to lactating mothers^41,42^. Freed et al^41^ surveyed pediatric residents and practitioners about breastfeeding knowledge and found that both groups had considerable knowledge deficits, and were ill-prepared for counseling mothers with lactation difficulties. Many residents surveyed in a study performed by Hillenbrand et al believed that early supplementation was not a cause of breast-feeding failure^43^, although previous studies have clearly demonstrated this relationship^44,45^. In the Ottawa study^17^, only 3.9% of women reported receiving breastfeeding help from the treating physician while admitted to the hospital, suggesting the possibility that physicians do not have adequate knowledge to assess proper breastfeeding methods and counsel appropriately.

Standards in providing breastfeeding assistance to mothers and children worldwide are well established. The International Board of Lactation Consultant Examiners (IBLCE) ensures competence in lactation consultant through the use of certification examination for lactation consultants^46^. A panel of experts from a variety of health professions, backgrounds, and geographic locations determined the dimensions of knowledge and skills necessary for safe and effective lactation consultant practice when the organization was founded. The guidelines are now continually reviewed and updated. The IBLCE was first accredited by the NCCA (National Commission for Certifying Agencies) in 1988, and has been reaccredited every five years since.

In summary, there is a need for this comprehensive study examining the impact of a breastfeeding support intervention for mothers of infants admitted to the hospital with jaundice. This study will also clarify the rates of early breastfeeding discontinuation in the jaundiced population, as well as help determine whether physicians in the Ottawa population have knowledge deficits when it comes to breastfeeding, as there may prove to be a role for incorporating teaching of breastfeeding skills to physicians and physicians in training.

**3. REFERENCES**

1. American Academy of Pediatrics. Breastfeeding and the Use of Human Milk. Policy Statement. Pediatrics 2005;115:496-506.
2. Heinig MJ. Host defense benefits of breastfeeding for the infant. Effect of breastfeeding duration and exclusivity. Pediatr Clin North Am. 2001;48:105-123, ix.
3. Horwood LJ, Fergusson DM. Breastfeeding and later cognitive and academic outcomes. Pediatrics. 1998;101 (1).
4. Anderson JW, Joshntone BM, Remley DT. Breastfeeding and cognitive development: a meta-analysis. Am J Clin Nutr. 1999;70:525-535.
5. Newcomb PA. Storer BE, Longnecker MP, et al. Lactation and a reduced risk of premenopausal breast cancer. N Engl J Med. 1994; 330: 81-87.
6. Collaborative Group on Hormonal Factors in Breast Cancer. Breast cancer and breastfeeding: collaborative reanalysis of individual data from 47 epidemiological studies in 30 countries, including 50302 women with breast cancer and 96973 women without the disease. Lancet. 2002; 360:187-195.
7. Rosenblatt KA, Thomas DB. Lactation and the risk of epithelial ovarian cancer. WHO Collaborative Study of Neoplasia and Steroid contraceptives. Int J Epidemiol 1993; 22: 192-197.
8. UNICEF-WHO. Innocenti Declaration. On the Protection, Promotion and Support of Breast-feeding. New York, NY:UNICEF;1990.
9. Bertini G, Dani C, Tronchin M, Rubaltelli FF. Is Breastfeeding Really Favoring Early Neonatal Jaundice? Pediatrics. 2001;107:e41.
10. Maisles MJ, Giffor K. Neonatal jaundice in full-term infants. Am J Dis Child. 1983;137:561-562.
11. Linn S, Schoenbaum SC, Monson RR et al. Epidemiology of neonatal hyperbilirubinemia. Pediatrics. 1985;75:770-774
12. Schneider AP. Breast milk jaundice in the newborn. JAMA. 1986;255:3270-3274.
13. Rubaltelli FF. Unconjugated and conjugated bilirubin pigments during perinatal development IV: the influence of breastfeeding on neonatal hyperbilirubinemia. Biol Neonate. 1993;64:104-109.
14. Nielsen HE, Haase P, Blaabjerg, et al. Risk factors and sib correlation in physiological neonatal jaundice. Acta Paediatr Scand. 1987;76:504-11.
15. Spatz DL, Goldschmidt KA. Preserving breastfeeding for the rehospitalized infant: a clinical pathway. MCN, Am J of Maternal Child Nursing. 2006;31:46-51.
16. Willis SK, Hannon PR, Susan C. The impact of the maternal experience with a jaundiced newborn on the breastfeeding relationship. J Fam Pract. 2002;51:465.
17. Pound CM, Gaboury I. The impact of jaundice in the newborn on length of breastfeeding. Paediatrics & Child Health. *.* 2009(7);14:445-9.
18. Guise JM, Palda V, Westhoff C, et al. The Effectiveness of Primary Care-Based Interventions to Promote Breastfeeding: Systematic Evidence Review and Meta-Analysis for the US Preventive Services Task Force. Ann Fam Med. 2003;1:70-80.
19. Kramer MS, Chalmers B, Hodnett ED et al. Promotion of Breastfeeding Intervention Trial (PROBIT). A Randomized Trial in the Republic of Belarus. JAMA. 2001;285:413-420.
20. Canadian Paediatric Society, Dietitians of Canada and Health Canada. Nutrition for Healthy Term Infants. 1998. Ottawa: Minister of Public Works and Government Services, 1998.
21. Health Canada. Exclusive Breastfeeding Duration: 2004 Health Canada Recommendation. Ottawa, 2004.
    [www.hc-sc.gc.ca/hpfb-dgpsa/onpp-bppn/exclusive_breastfeeding_duration_e.html](http://www.hc-sc.gc.ca/hpfb-dgpsa/onpp-bppn/exclusive_breastfeeding_duration_e.html).
22. World Health Organization. Global Strategy for Infant and Young Child Feeding, The Optimal Duration of Exclusive Breastfeeding. Geneva, 2001. [www.who.int/gb/ebwha/pdf_files/WHA54/ea54id4.pdf](http://www.who.int/gb/ebwha/pdf_files/WHA54/ea54id4.pdf)
23. Cochi SL, Fleming DW, Hightower AW, et al. Primary invasive *Haemophilus influenzae* type b disease: a population-based assessment of risk factors. J Pediatr. 1986;108:887-896.
24. Istre GR, Conner JS, Broome CV, Hopkins RS. Risk factors for primary invasive *Haemophilus* influenzae disease: increased risk from day care attendance and school-aged household members. J Pediatr. 1985;106:190-195.
25. Takala AK, Eskola J, Palmgren J, et al. Risk factors of invasive *Haemophilus influenzae* type b disease among children in Finland. J Pediatr. 1989;115:694-701.
26. Dewey KG, Heinig MJ, Nommsen-Rivers LA. Differences in morbidity between breast-fed and formula-fed infants. J Pediatr. 1995;126:696-702.
27. Howie PW, Forsyth JS, Ogston SA, et al. Protective effect of breastfeeding against infection. BMJ. 1990;300:11-16.
28. Popkin BM, Adair L, Akin JS, et al. Breastfeeding and diarrheal morbidity. Pediatrics. 1990;86:874-882.
29. Lopez-Alarcon M, Villalpando S, Fajardo A. Breastfeeding lowers the frequency and duration of acute respiratory infection and diarrhea in infants under six months of age. J Nutr. 1997;127:436-443.
30. Bachrach VR, Schwarz E, Bachrach LR. Breastfeeding and the risk of hospitalization for respiratory disease in infancy: a meta-analysis. Arch Pediatr Adolesc Med. 2003;157:237-243.
31. Oddy WH, Sly PD, de Klerk NH, et al. Breastfeeding and respiratory morbidity in infancy: a birth cohort study. Arch Dis Child. 2003;88:224-228.
32. Duncan B, Ey J, Holberg CJ, Wright AL, et al. Exclusive breastfeeding for at least 4 months protects against otitis media. Pediatrics. 1993;91:867-872.
33. Aniansson G, Alm B, Andersson B, et al. A prospective cohort study on breastfeeding and otitis media in Swedish infants. Pediatr Infect is J. 1994;13:183-188.
34. Marils S, Hansson J, Jodal U, et al. Protective effect of breastfeeding against urinary tract infection. Acta Paediatr. 2004;93:164-168.
35. Pisacane A, Graziano L, Mazzarella G, et al. Breastfeeding and urinary tract infection. J Pediatr. 1992;120:87-89.
36. Paricio Talayero JM, Lizan-Garcia M, Otero Puime A, et al. Full breastfeeding and hospitalization as a result of infections in the first year of life. Pediatrics 2006; 118:e92.
37. Ottawa Public Health. “Infant Care Survey 2005”. Ottawa, Ontario, 2006
38. Ahluwalia IB, Morrow B, Hsia J. Why do women stop breastfeeding? Findings from the Pregnancy Assessment and Monitoring System. Pediatrics 2005;116:1408.
39. Dunn S, Davies B, McCleary L, et al. The Relationship Between Vulnerability Factors and Breastfeeding Outcome. JOGNN. 2006;35:87-97.
40. Britton C, McCormick FM, Renfrew MJ, et al. Support for breastfeeding mothers (Review). Cochrane Database of Systematic Reviews 2007, Issue 1. Art. No.: CD001141.
41. Freed GL, Clark SJ, Sorenson J, Lohr JA, Cefalo R, Curtis P. National assessment of physicians’ breast-feeding knowledge, attitudes, training, and experience. JAMA. 1995;273:472-476.
42. Williams EL, Hammer LD. Breastfeeding attitudes and knowledge of pediatricians-in-training. Am J Prev Med. 1995;11:26-33.
43. Hillenbrand KM, Larsen PG. Effect of an Educational Intervention About Breastfeeding on the Knowledge, Confidence, and Behaviors of Pediatric Resident Physicians. Pediatrics. 2002;110:e59.
44. Olson CM, Psiaki DL. Imparting information on breastfeeding to medical students. J Med Educ. 1978;53:845-847.
45. Wright A, Roce S, Wells S. Changing hospital practices to increase the duration of breastfeeding. Pediatrics. 1996;97:669-675.
46. [http://www.iblce.org](http://www.iblce.org/)
47. Moher D, Schulz KF, Altman DG. The CONSORT statement: revised recommendations for improving the quality of reports of parallel-group randomized trials. Lancet 2001;357:1191-1194.
48. [Breastfeeding best practice guidelines for nurses.](http://www.guideline.gov/summary/summary.aspx?doc_id=11506&nbr=005959&string=breastfeeding) Registered Nurses' Association of Ontario - Professional Association.  2003 Sep (addendum released 2007 Mar)
49. Clinical Practice Guideline. Management of Hyperbilirubinemia in the Newborn Infant 35 or more weeks of gestation. Pediatrics 2003;114:297-316.
50. Sadeharju K, Knip M, Virtanen SM, et al. Maternal antibodies in breast milk protect the child from enterovirus infections. Pediatrics. 2007;119:941-946.
51. Wright AL, Holberg CJ, Martinez FD, et al. Group Health Medical Associates. Breast feeding and lower respiratory tract illness in the first year of life. BMJ. 1989; 299:946-949.
52. Von Kries R, Koletzko B, Sauerwald T et al. Breast feeding and obesity; cross sectional study. BMJ. 1999; 319:147-150.
53. Krousel Wood MA, Re RN, Abdoh A et al. The effect of education on patients’ willingness to participate in a telemedicine study. J Telemed Telecare. 2001;7:281-287.
54. Even C, Richard H, Thuile J et al. Characteristics of voluntary participants versus nonparticipants in a psychoeducation program for euthymic patients with bipolar disorder. J Nerv Ment Dis. 2007;195:262-265.
55. Rimer BK, Schildkraut JM, Lerman C, et al. Participation in womens’ breast cancer risk counseling trial. Who participates? Who declines? High Risk Breast Cancer Consortium. Cancer. 1996;77:2348-2355.
56. Plint AC, Johnson DW, Wiebe N, et al. Practice variation amoung pediatric emergency departments in the treatment of bronchiolitis. Acad Emerg Med. 2004;11:353-360.
57. Plint AC, Osmond MH, Klassen TP. The efficacy of nebulized racemic epinephrine in children with acute asthma: a randomized, double-blind trial. Acad Emerg Med. 2000;10:1097-1103.
58. Patton (2002) Qualitative evaluation and research methods, 3^rd^ ed. Los Angeles CA: Sage.
59. Plint AC, Johnson DW, Patel H et al. Epinephrine and dexamethasone in children with bronchiolitis. N Engl J Med. 2009; 360: 2079-89

# 4. RESEARCH DESIGN

**4.1 Trial Design:** This will be a randomized controlled trial with a qualitative component. Blinding of the participants in each study group will not be feasible, as the intervention group will receive counseling from a lactation consultant and the control group will not. The investigators, data analyst, and research assistant completing study follow-up will be blinded to group assignment.

**4.2 Population:** Mothers of all breastfeeding infants admitted to the inpatient units of the Children’s Hospital of Eastern Ontario (CHEO) with jaundice will be considered for this study. CHEO is a tertiary care centre that provides pediatric care to a population of 1.5 million people.

**4.3 Eligibility Criteria**

***Inclusion Criteria***

-Mothers of infants admitted during the study period with hyperbilirubinemia, breastfeeding at the time of admission (any amount of breastfeeding)

-Mothers of infants < 1 month of age at the time of admission

***Exclusion criteria***

-Mothers of infants admitted with hyperbilirubinemia who are exclusively formula-fed

-Mothers of infants with hyperbilirubinemia of the predominantly conjugated type as this is a different disease, not associated with breastfeeding difficulties

-Mothers of infants with anatomical abnormalities, such as cleft lip or palate, as this would interfere with breastfeeding and require more intensive intervention.

-Mothers of neurologically impaired infants as breastfeeding may be more difficult in this population

-Mother of infants who were admitted to the NICU after birth and never went home as they are likely to have other comorbidities affecting feeding.

-Mothers of infants feeding via naso-gastric, naso-jejunal, or gastric tube

-Mothers of infants > 1 month of age

-Mothers who have had breast surgery in the past

-Foster mothers or adoptive mothers

-Mothers who do not understand English or French

-Mothers of infants that are the result of multiple birth (eg twins)

**4.4 Sample selection**

All mothers of breastfeeding infants admitted to hospital with hyperbilirubinemia and fitting the inclusion criteria will be approached and screened for eligibility. A research assistant will review the list of admissions for the previous 24 hours, on a daily basis, from Tuesday to Friday, and for the previous 72 hours on Monday morning in order to ensure that all infants admitted to the hospital with hyperbilirubinemia are considered. The research assistant will then ask a member of the patient’s health care team (bedside nurse, charge nurse, resident, staff physician) to ask the family for permission to approach them about the study. The research assistant will then complete an eligibility screen (Appendix 1). If the patient is eligible and the family gives their informed consent (Appendix 2), they will be allocated to one of the study groups. If the family is allocated to the lactation consultant, the consultant will then be contacted.

For patients admitted after hours on Friday, or during the weekend hours, and that are expected to be discharged home before Monday morning, the attending physician responsible for the patient will approach the family and complete the eligibility screen. If the patient is eligible and the family gives informed consent, the patient will be allocated to one of the study groups. If the family is allocated to the lactation consultant, the consultant will then be contacted and will come in for the initial consultation prior to discharge. A message will be left on the research assistant’s voice mail, who will then follow-up when she returns on Monday morning. A record will be kept of all screened patients, patient eligibility, allocation, and follow-up to allow reporting according to CONSORT guidelines^47^.

**4.5 Randomization and Allocation Concealment**

After enrollment, the patients will be randomly allocated to one of the two treatment groups. The randomization schedule will have been previously generated using a computer. Randomization will be blocked with randomly chosen block lengths of 4 or 6. Treatment assignments will be written on a piece of paper and concealed in sequentially numbered opaque envelopes kept in a secure locked location in the study research office. The PI and analyst will be blinded to the treatment intervention, but the lactation consultant and research assistant in charge of assessing eligibility and allocation will not because of the nature of the experimental intervention, which consists of meeting with a lactation consultant.

**4.6 Study Interventions**

***Intervention group***

Mothers randomized to the intervention group will meet with an International Board of Lactation Consultant Examiners (IBLCE)-certified lactation consultant once during the infant’s hospitalization. The lactation consultant’s intervention will be based on established clinical practice guidelines^48^ (Appendix 3). The lactation consultant will review the benefits of breastfeeding with the mother. Assessment of breastfeeding techniques will be performed, and corrected as necessary. Mothers will be taught how to use a breast pump if the lactation consultant feels a breast pump is necessary, and specific guidelines as to when to use the pump will be discussed. Mothers will also be instructed on how to safely store their breast milk for later use. Breast pumps are already available in the hospital for breastfeeding women. Breast pumps, containers for milk storage, bottles and nipples will be provided to women who need them at no cost. Breast pumps will be loaned for a period of 6 weeks, after which the pumps will have to be returned.

Once the infant has been discharged from the hospital, the lactation consultant will offer three weekly half-hour follow-up sessions at the Children’s Hospital of Eastern Ontario (CHEO) (see Appendix 3 for further details ). The follow-up sessions will be available during day and evening hours. At every follow-up visit, the baby will be weighed on the same scale to aid in assessment of feeding difficulties. Breastfeeding techniques will again be reviewed and corrected as necessary, and the mother’s questions or concerns will be addressed. In the event that a problem is noted, such as weight loss, jaundice, decreased arousal, or any other symptom that is of concern to the lactation consultant, the child will be sent to the emergency department if urgent intervention is needed and the PI will be notified within 24 hours. The lactation consultant will keep a file for each patient, in which she will document the details of each encounter.

Once the follow-up sessions are over, the lactation consultant will provide the mother with a list of resources available should further breastfeeding issues arise in the future. There will be 2 lactation consultants available for the whole study duration. The lactation consultant’s call schedule will be randomly generated by computer. Each lactation consultant will provide follow-up for the patients they initially see, unless unforeseen circumstances such as illness occur. We are ensuring standardization of the intervention procedure by having the lactation consultants generate a tool they will use to assess the families.

***Control group***

Mothers randomized to the control group will receive the current standard of medical care for hyperbilirubinemia at the CHEO (i.e., phototherapy and intravenous fluids as described below). There is no current standard intervention in terms of breastfeeding support. A mother may benefit from advice given by the nurses if the nurse assigned to her child has breastfeeding knowledge. This is not however standardized, and recommendations vary from person to person. Mothers who participated in our previous study reported a wide range of attitudes towards breastfeeding coming from all health care professionals, ranging from no support at all, or even recommendations against breastfeeding, to very supportive^17^.

Because there are private lactation consultants, as well as public health nurses that are available in the City of Ottawa through self-referral, it is possible that women in the control group will seek breastfeeding support once discharged from the hospital. This information will be recorded in the booklet provided to the study participants (see Appendix 4) as well as during phone follow-ups. This information will be available when analyzing the study data, allowing the authors to determine possible contamination between groups. Despite the fact that these resources are widely available in the city, our previous study demonstrated that approximately half of patients admitted to the hospital with jaundice did not seek any breastfeeding help, and of those who did, the amount and type of help received differed widely from person to person.

Given that the purpose of this study is to show the benefit of a hospital-based breastfeeding support intervention over current standard of care, and that self referral to community based support programs is part of the standard of care and cannot be controlled for, the investigators feel it is important to allow women in the control group to seek help as they deem it appropriate.

***Treatment of hyperbilirubinemia***

All infants will receive care for hyperbilirubinemia as determined by the treating physician. This treatment typically consists of phototherapy, intravenous fluids, and monitoring of the infant’s bilirubin levels. Therapy is guided by the child's bilirubin level as plotted on a nomogram^49^ (see Appendix 5). The level at which phototherapy is discontinued depends on age and risk factors of the child (gestational age, hematological disorder, concomitant sepsis, anoxia at birth, etc) and is determined by the nomogram. Once phototherapy is discontinued, intravenous fluids are weaned off and bilirubin is then monitored off treatment. Infants are usually discharged home once their bilirubin is below the threshold for phototherapy, as determined by the nomogram, and off intravenous fluids.

**4.7 Study Measurements:**

Baseline information about the infant and the infant’s mother will be recorded at the time of study enrollment. Information about the mother will include language spoken (French or English), maternal age, number of children prior to the participating infant, number of previously breastfed children, maternal education level, household income, marital status, maternal smoking history during pregnancy and after delivery, type of health care provider who followed the pregnancy, attendance at pre-natal classes. Information about the infant will include age, birth weight, gestational age at birth, weight on admission, weight on discharge, need for IV therapy during the hospitalization, septic work up done on admission, antibiotic treatment, length of stay, and the need for formula supplementation before admission, as well as during hospital stay (see Appendix 6).

Follow-up measurements will be collected until the infant is six months of age. Mothers will be given a booklet upon discharge from the hospital, where they will be asked to record the information on formula introduction, solid food introduction, timing of return to work, number of daily formula feeds and sessions at the breast at different points in time, number of hospitalizations and reason, number of visits to primary care physicians and reason, discussion with primary care physician regarding breastfeeding, number of visits to breastfeeding support (see Appendix 6).

A second research assistant, blinded to group allocation, will phone the participating mothers 1 week after discharge from the hospital to remind them to fill out their booklet, and to collect information regarding breastfeeding and formula feeding. Other phone calls will be placed again when the infant is 2, 3, 4 and 6 months old, to remind the mothers to fill out their booklets. The research assistant will also collect the same information the women are asked to record in their booklet, as a back up method in case the booklets are lost or not appropriately filled out. The research assistant will also remind the families, at the beginning of the phone call, not to disclose whether they were allocated to the control or to the study group so that the research assistant can remain blinded.

Feedback on the intervention will be obtained via semi-structured phone interviews. Twenty women from the intervention group will be randomly selected. Consent to contact the mothers for the purpose of these interviews will have been obtained on the original consent form signed at enrollment. A first set of phone interviews will occur at the end of the intervention period (after the 3 follow-up sessions with the lactation consultant are completed), and at the end of the study period (after the 6 month follow-up period). The interview tool (see Appendix 7) will elicit information on the participants’ perceptions of the intervention. More specifically, what they found most and least helpful, and what changes they would like to see if a breastfeeding support program were to be implemented at the hospital.

Semi-structured phone interviews will also be conducted with women from the control group to gather information on their experience at CHEO, and to determine whether they believe they received adequate support with breastfeeding (see Appendix 8). Twenty women from the control group will be randomly selected for interviews. Consent to contact the mothers for the purpose of these interviews will have been obtained on the original consent form signed at enrollment. A first set of phone interviews will occur one week after hospital discharge, and at the end of the study period (after the 6 month follow-up period).The semi-structured interviews will be conducted for both groups by the first research assistant, as that person will not be blinded to group allocation as he / she will be the person contacting the lactation consultant for mothers allocated to the intervention group.

At the end of the study period, an International Board Certified Lactation Consultant, independent of the study, will be asked to review 10% of the files kept by the study lactation consultant to ensure that advice provided to women met standard of care, as defined by the International Board of Lactation Consultants.

**4.8 Outcomes:**

***Primary:*** The primary outcome measured will be exclusive breastfeeding at 3 months, or 3 months corrected if the infant was born prematurely. Exclusive breastfeeding is defined as no other milk intake than maternal milk. Health benefits have been demonstrated after as little as two weeks of exclusive breastfeeding^50^. However, a larger number of studies have reported positive health outcomes after two to four months of exclusive breastfeeding^27, 50-52^. Exclusive breastfeeding was chosen as primary outcome as it is a quantifiable measure. Partial breastfeeding is more difficult to define as it may range from very little breastfeeding to almost exclusive breastfeeding, which would have an impact on the magnitude of benefits imparted by breastfeeding. Breastfed babies will be defined as babies who receive no other milk than breast milk, whether directly from the breast or as expressed breast milk.

***Secondary:*** All measurements will be collected at corrected age if infant was born prematurely. 1) Exclusive breastfeeding at six months; 2) Partial breastfeeding at three months; 3) Partial breastfeeding at six months; 4) Number of re-hospitalization for jaundice in the first six months of life; 5) Number of re-hospitalization for non-jaundice related causes in the first six months of life; 6) Number of physician encounters in first 6 months of life; 7) Number of mothers seeking breastfeeding help; 8) Amount and type of breastfeeding support given by child’s primary physician in first six months of life

# 5. ANALYSIS

**5.1 Sample size:**

In our previous study of infants hospitalized for jaundice, the rate of exclusive breastfeeding at 3 months was roughly 50%^17^. However, we expect that rate to be lower given the reasons stated above (high level of education in the study population). Moreover, higher levels of education have been shown to be associated with higher rates of participation in different types of study^53-55^, making the selection bias hypothesis even more likely. Nevertheless, to be conservative we picked 50% as the rate of exclusive breastfeeding at 3 months.

Based on a systematic review of interventions to promote breastfeeding, a 25% increase in the rate of breastfeeding at 3 months is plausible^18^. In that meta-analysis of randomized controlled trials combining educational and support programs, breastfeeding rates in the short term increased by 36% (95% CI 22-49%), putting our 25% increase estimate at the low end of the confidence interval. This would result in an additional one in four women breastfeeding. An informal survey of hospital based consultant pediatricians confirms this as a clinically significant increase. Setting the probability of type I error at 0.05, a sample size of 58 mothers per group would result in a power of 80% to detect such an increase.

The anticipated number of patients lost to follow-up for the primary outcome is low given that our population of interest consists of young infants. Previous studies in such young children conducted at CHEO have high follow-up rates when primary outcomes were determined through phone follow-up^56,57^. As a result, we expect a loss to follow-up of no more than 7.5%. To account for this, we would therefore need to recruit 62 patients in each group (Appendix 9).

**5.2 Quantitative Data Analysis**

Baseline characteristics of the study participants will be summarized using descriptive statistics. Continuous variables will be summarized using the mean, median, standard deviation, interquartile range, and range. Discrete variables will be summarized using frequencies and percentages.

The primary analysis will be a comparison between groups of breastfeeding rates at 3 months using Fisher's exact test. All tests will be two-sided, with p-values less than 0.05 taken to be statistically significant. Time to discontinuation of breastfeeding will also be compared between groups graphically using Kaplan-Meier curves, and statistically using the logrank test. Mothers still breastfeeding at the time of data collection will be treated as censored observations. The rates of breastfeeding failure (defined as discontinuation of breastfeeding before the infant is 6 months old) will be compared between mothers of infants admitted to the hospital with jaundice and randomized to the control group, and those in the community as per the breastfeeding rates determined by the Infant Care Survey^37^ using Pearson’s chi-squared test. Poisson regression models will be used to compare the number of re-hospitalizations for jaundice, hospitalizations for non-jaundice related causes, as well as physician encounters (emergency room visits, community pediatricians / family physicians) in both groups of patients throughout their first 6 months of life. Fisher’s exact test will be used to compare the proportion of mothers in each group seeking breastfeeding help once discharged from the hospital.

**5.3 Qualitative Data Analysis**

All semi-structured interviews will be audio taped and transcribed verbatim. The transcription of the audiotapes will allow for accurate reporting of the responses and for interpretation of responses within the context of the entire discussion. Each participating mother will be provided with the opportunity to verify the transcript of her interview. This verification will ensure the quality and accuracy of the data. All qualitative data analyses will be conducted using Nvivo 8 software. Inductive analysis will be used to identify categories, patterns and themes that are embedded in the data and seem meaningful to the interviewees^58^.

**5.4 Feasibility**

In 2006, there were 116 breastfeeding infants admitted with jaundice to the Children’s Hospital of Eastern Ontario who met the eligibility criteria of this study, as determined by the CHEO Health Record Analyst. Given that the average length of stay for these patients was 2.5 days, we estimate that 90% of the families admitted to the hospital will be approached, and that 80% of those will consent to participate in the study, for a total enrolment rate of 72%. Based on these numbers, the period of recruitment will last 18 months to achieve our total sample of 124, for a total study duration of 2 years.

Our 90% approaching rates are based on strong strategies put in place to avoid missing eligible patients. As mentioned above, the average length of stay is 2.5 days for babies admitted with jaundice, with very few babies staying less than 24 hours. The admission log will be checked every morning from Monday to Friday by the research assistant, and eligible families will be approached. On Friday afternoon, the research assistant will leave 2 to 3 opaque envelopes in a secure location on the wards. On Saturday and Sunday mornings, the attending physicians responsible for the inpatient units will determine if any of the babies admitted over the previous 24 hours are eligible for the study Eligible families will then be approached by the attending physician on that day. If the family agrees to participate, the attending physician will open the opaque envelope, and communicate with the lactation consultant if randomized to the intervention group. The family will then meet with the lactation consultant if randomized to the intervention group, since the lactation consultant will be available 7 days a week. The attending physician will then be instructed to leave a voice mail message with the research assistant, so that follow-up can be ensured. On Monday morning, the research assistant will pick up the unopened envelopes from the secure location. One of the investigators (CP, AP) will be available by pager to answer the attending physician’s questions, if any. If we find that less than 90% of families are being approached, other strategies will be put in place, such as 24 hours paging of the investigators and/or research assistant by the admitting clerk.

Previous studies done at CHEO with more aggressive interventions were shown to have consent rates of 60%^59^. These studies looked at drug intervention therapies in patients presenting to the emergency department, a highly stressed population. Given that our intervention (breastfeeding support offered by a lactation consultant) presents no risk at all, and our population is highly motivated, a consent rate of 80% seems highly reasonable. Also, in the PI’s retrospective study examining length of breastfeeding in jaundiced patients, over 60% of mothers who were mailed a questionnaire participated in the study, showing that this population is highly motivated^17^. Given that our intervention (breastfeeding support offered by a lactation consultant) presents no risk at all, and our population is highly motivated, a consent rate of 80% seems highly reasonable.

# 6. TRIAL MANAGEMENT

# 6.1 Day to day management

The principal investigator (CP) and study coordinator will be responsible for the overall supervision of the study. A first study site research assistant will be responsible for recruiting patients, allocating patients to their intervention, communicating with the lactation consultant when patients are randomized to the intervention group, and conducting the semi-structure interviews. A second research assistant, blinded to group allocation, will collect and enter the data, and provide phone follow-ups one week after discharge, and at 2, 3, 4, and 6 months. An International Board-Certified Lactation Consultant (IBLC) will provide the breastfeeding support intervention.

Data entry and management will be provided by Clinical Research Group of the Children’s Hospital of Eastern Ontario Research Institute. The statistician (NB) will be responsible for statistical data analysis and will assist in manuscript preparation. ACP and RC will provide support to the PI and study coordinator in implanting the study. KF and KM will provide guidance in the qualitative component of the study.

Breastfeeding support equipment (breast pumps, collection kits, nipple shields, freezer bags, nursing pads) will be provided at lower cost by Medela Canada Inc. A letter detailing cost of supplies is attached (Appendix 10). The company’s sole involvement with the study is the provision of breastfeeding equipment. They will not be involved in the analysis, interpretation, or writing of the final manuscript.

# 6.2 Study Team

The team includes experts in pediatric medicine (CP, ACP, KF), epidemiology (ACP, NB), qualitative studies (KF, KM), biostatistics (NB), and clinical trial implementation (RC). Dr Pound is a new investigator, but has expertise in the breastfeeding field as demonstrated by her previous work mentioned above. Dr. Plint has extensive research experience and has completed randomized controlled trials in the area of pediatrics. Dr. Farion has experience in clinical trials and qualitative studies. Dr Barrowman is an established statistician with broad experience in clinical trial design. K Moreau has expertise in evaluation and mixed-methods. R Correll is a highly experienced research coordinator of multicentre trials

Two International Board-Certified Lactation Consultants (IBLC) have been recruited. Ms Susan Lepine is currently employed as a registered nurse in the Neonatal Intensive Care Unit at CHEO, and is one of the Ottawa Perinatal Coordinators for the Perinatal Partnership Program of Eastern and Southeastern Ontario (PPPESO) (see letter of support Appendix 11). She has extensive experience in the area of breastfeeding support. Ms Gisele Gobeil is a registered nurse, as well as a lactation consultant, childbirth educator, birth and postpartum doula. She has extensive experience in the areas of breastfeeding support and education.

# 7. ETHICAL CONSIDERATIONS

Patients will only be entered into the study after free and informed consent (Appendix 2) has been received from the mother. In addition, they will be free to withdraw at any point in time and will receive the same standard of medical care given to all jaundiced patients admitted to the pediatric ward at CHEO. The data collected during this trial will be held confidential.

The intervention in this trial will be unlikely to have any adverse effect on the patient or patient’s mother, as there is no risk associated with filling out a questionnaire, a booklet, and meeting with a lactation consultant. There will be no discomfort to the patient as all the interventions are done with the mother. Those mothers from the intervention arm randomly approached to participate in the follow-up interviews will be asked to consent to their interview being audio-taped and transcribed for ease of analysis. They will be assured that their thoughts and opinions will not be linked to them in any way during the analysis or presentation of results.

We do not feel that it is unethical to withhold the service of a lactation consultant for the control group as this is the current standard of care at CHEO. There is no study in the literature examining the impact of a breastfeeding support intervention on jaundiced infants admitted to hospital. Given the clear advantages that breastfeeding confers to the health of children, it is reasonable to conduct a clinical trial to determine the effectiveness of an intervention as we have described.

# 8. RELEVANCE OF STUDY AND DISSEMINATION FO RESULTS

The results of this study will clarify the importance of offering sound breastfeeding advice and accessible resources to mothers of young infants admitted to the hospital with jaundice, thus determining whether there truly is a need for trained lactation specialists in pediatric hospitals. It will further allow us to determine whether this particular population is truly at risk for early breastfeeding discontinuation. It will also allow us to determine whether a hospital-based breastfeeding support program can have a quantifiable impact on children’s health in their first 6 months of life, as measured by physician encounters and number and length of hospitalization. It will also help determine whether the implementation of a breastfeeding support program in the hospital will have an impact on the number of women seeking breastfeeding help post discharge. Lastly, it will allow us to collect information on the amount of support that is provided to breastfeeding women by their child’s primary care physician, potentially identifying a need for more rigorous breastfeeding training during residency.

The results of this study will be submitted for publication in peer-reviewed journals and submitted for presentation at appropriate scientific meetings. Results will also be provided to the heads of the academic health centres, to the hospitalist section of the Canadian Pediatric Society, and to the International Lactation Consultant Association (ILCA). They will pave the grounds for larger scale studies, with diverse infant populations.

**Appendix 1**

**Screening Log**

| **Screening #** | **Medical Record Number** | **Date of Visit**  **(dd/mm/year)** | **Eligible**  **for Study** | **Enrolled**  **in Study** | **If YES,**  **Enrollment**  **study #** | **If No, Why?**  **Circle**  **Reason** | **Signature** |
| --- | --- | --- | --- | --- | --- | --- | --- |
|  |  |  | **Y / N** | **Y / N** |  | 1-2-3 |  |
|  |  |  | **Y / N** | **Y / N** |  | 1-2-3 |  |
|  |  |  | **Y / N** | **Y / N** |  | 1-2-3 |  |
|  |  |  | **Y / N** | **Y / N** |  | 1-2-3 |  |
|  |  |  | **Y / N** | **Y / N** |  | 1-2-3 |  |
|  |  |  | **Y / N** | **Y / N** |  | 1-2-3 |  |
|  |  |  | **Y / N** | **Y / N** |  | 1-2-3 |  |
|  |  |  | **Y / N** | **Y / N** |  | 1-2-3 |  |
|  |  |  | **Y / N** | **Y / N** |  | 1-2-3 |  |
|  |  |  | **Y / N** | **Y / N** |  | 1-2-3 |  |
|  |  |  | **Y / N** | **Y / N** |  | 1-2-3 |  |

Legend : 1- Research Assistant not available

2- Family refused enrollment

3- Other

**Appendix 2**

**Consent form for patient’s participation**

**Patient Study Number:______________**

**The Impact of a Breastfeeding Support Intervention on Breastfeeding Duration in Jaundiced Infants Admitted to a Tertiary Care Centre; a Randomized Controlled Trial.**

Women are being invited to participate in a study.

This study is being done by the Children’s Hospital of Eastern Ontario (Ottawa).

The principal investigator doing this study is:

- Catherine Pound (pediatrician)

Dr Pound can be contacted at 613-737-7600 ext 2571 for questions related to the study.

The co-investigators are:

- Amy Plint
- Ken Farion
- Katherine Moreau
- Nick Barrowman
- Rhonda Correll

**Why is this study being done?**

Research has suggested that babies admitted with jaundice to the hospital may not breastfeed as long as other babies. Women whose babies are admitted to hospital for jaundice may find it difficult to continue to breastfeed exclusively after taking their babies home. Research has shown, however, that breastfeeding is good for babies and has clear benefits to their health. Specifically, breastfed babies have been shown to be healthier in their first year of life, and do better on neurocognitive tests many years down the line. The purpose of this study is to see whether or not breastfeeding support helps women maintain breastfeeding after discharge from the hospital. In this study, women will be randomly assigned to see a ‘lactation consultant’. A lactation consultant is an individual with special training in breastfeeding support. We will assess the impact of this intervention on the length of breastfeeding, the health of the children during their first 6 months of life, as well as the difference in breastfeeding duration between babies who receive help from the lactation consultant and those who do not. We will also collect information on mothers who seek breastfeeding advice when discharged from the hospital, and what kind of support they get from their child’s primary care physician. Currently, there are no formally trained lactation consultants available to mothers whose infants are admitted to the hospital. Mothers with breastfeeding problems may ask for their nurse’s or doctor’s help, however he / she may not have received any formal training in breastfeeding support.

**How many people will take part in this study?**

124 babies under the age of 1 month at the time of admission to hospital and their mothers will be recruited to participate in this study.

**What is involved in the study?**

You and your child’s personal information will be recorded when you accept to participate in the study (your age, number of children before this one, number of breastfed children before this one, education level, household income, marital status, smoking, planned date of return to work, your child’s age at admission to hospital, gestational age at birth, birth weight, weight on admission to hospital, weight on discharge from hospital, and investigations and treatments done while in hospital).

You will be given a booklet to fill out on discharge from the hospital The booklet will be used to collect information on your baby’s feeding, his or her health, the number of times you saw a physician, and the type of breastfeeding advice that was given to you by your physician, other health care professionals, friends or family.

Someone will also contact you one week after your child is discharged, and when your child is 2, 3, 4 and 6 months to remind you to fill out the booklet and ask you the same questions that are in the booklet. This will serve as a back up method to record your information if something happens to the booklet. This phone call will take 20 minutes of your time.

Also, if you agree to it, you may receive two extra phone calls during the study period. This interview will collect information on your experience at CHEO, as well as your experience with the lactation consultant if you met with her. You may be contacted a few weeks after your discharge from the hospital, and again after your child is 6 months of age. This phone call will take 15 minutes to an hour of your time, depending on how much information you share with us. This conversation can be scheduled according to your schedule, or can be broken down in a few different sessions if it is more convenient for you.

If you agree to participate in the study, you and your child will be randomized into one of two groups; one will receive counseling from a lactation consultant, and the other will receive standard, routine support in the form of advice from staff at the hospital. Randomization means that you are put into a group by chance. You will have an equal, 1 in 2 chance of being placed in one of the groups. All babies will receive the usual medical care for jaundice (usually phototherapy and intravenous fluids) as suggested by their doctor. You and the research staff will not know which group you and your baby will be assigned to before you decide whether or not you will participate in the study.

If your child is assigned to the control group this is all that is involved from the study, unless you are selected to receive the extra phone call described above. Only twenty women in each group will receive the extra phone call, so you may not be contacted even if you agreed to it initially.

If your child is assigned to the intervention group, you will meet with a lactation consultant once while in the hospital. After discharge, the lactation consultant will meet with you, at the hospital, once per week for three weeks. Your parking expenses for these three follow-up visits at CHEO will be paid for. Each meeting will be 30 minutes long and will occur in the late afternoon or early evening (as you prefer) to review breastfeeding techniques and make sure everything is going well. The lactation consultant may recommend that you express your breast milk if she feels it would help increasing your milk supply. She will provide you with a pump and pumping equipment for up to six weeks. Again, if you agreed to the extra phone calls, you may be contacted as explained above.

Participation in this study is voluntary, and you are free to withdraw from the study at any time. There will be no penalty to you and your child.

**What are the risks of the study?**

There are no risks involved with this study since all that is required from the mothers is to fill out a booklet, answer questions over the phone, and meet with a lactation consultant if randomized to the intervention group. One possible inconvenience of the study is that women might feel pressured to continue breastfeeding their infant. The research team supports exclusive breastfeeding but will make every effort to respect your decision regarding feeding of your infant.

**Are there benefits to taking part in the study?**

The results of this study will help determine whether or not meeting with a lactation consultant helps women continue breastfeeding after their babies were hospitalized for jaundice. If your child is randomized to the intervention group, you will benefit from 4 sessions with a lactation consultant and may therefore receive help with your breastfeeding techniques.

**Are there other options?**

If you do not wish to participate in this study, you will continue to receive standard care by the ward pediatric team at the hospital. You will also be free to visit publicly funded lactation clinics, or to hire a private lactation consultant. The cost of a private lactation consultant ranges from 10$ to 90$ an hour in Ottawa. Information about breastfeeding in the city of Ottawa can be found on the City of Ottawa website; <http://www.ottawa.ca/residents/health/living/nutrition/birth_six/index_en.html> (in English) <http://www.ottawa.ca/residents/health/living/nutrition/birth_six/index_fr.html> (in French).

**What about confidentiality?**

We will respect your privacy. Your and your child’s personal information will be kept strictly confidential except as required or permitted by law. The research data will be kept for a period of 5 to 7 year, after which it will be destroyed. No identifying data will be published about you or your child. Your and your child’s results will be treated anonymously. Your and your child’s records will be read only by staff members caring for you and by study investigators who will be analyzing the research information. However, representatives from the CHEO Research Ethics Board can have access to your personal information as per CHEO hospital policy**.**

**What are my rights as a participant?**

Participation in this study is voluntary. No compensation for participation will be given. It is your choice to take part in this study. You can also withdraw from the study at any time. The care you receive will not be affected whether you decide to participate in the study or not.

This study has been reviewed and approved by the CHEO Research Ethics Board. The CHEO Research Ethics Board is a committee of the hospital that includes individuals from different professional backgrounds. The Board reviews all human research that takes place at the hospital. Its goal is to ensure the protection of the rights and welfare of people participating in research. The Board’s work is not intended to replace a parent or child’s judgment about what decisions and choices are best for them. You may contact the Chair of the Research Ethics Board, for information regarding patient’s rights in research studies at (613) 737-7600 (3272), although this person cannot provide any health-related information about the study.

At the conclusion of the study, you will be given a summary of the results if you so wish.

**Who do I call if I have questions or problems?**

You may call

Research Coordinator (to be named) ext:

Or Dr Catherine Pound at 613-737-7600

I hereby consent for me and my child to take part in this study.

I will receive a signed copy of this form

­­____________________________ ______________________ _____________

Name of patient’s mother Signature Date

____________________________ ______________________ _____________

Name of person who got consent Signature Date

____________________________ ______________________ _____________

Name of investigator Signature Date

Please sign your name below if you agree to be contacted shortly after discharge from the hospital, as well as at the end of the study period for a phone interview. This discussion would have the purpose of collecting your impressions on your stay at CHEO, and on which part of the intervention you found most helpful, if you received it.

­­­­_____________________________

**Appendix 3**

**Lactation Consultant’s Intervention**

**Initial consultation**

Assessment of:

- Level of maternal physical discomfort
- Observation of positioning, latching, and sucking
- Signs of milk transfer
- Parental ability to identify infant feeding cues
- Mother-infant interaction and maternal response to feeding cues
- Maternal perception of infant satisfaction/satiety cues
- Woman’s ability to identify significant others who are available and supportive of breastfeeding
- Maternal breastfeeding self-efficacy

Education on:

- Benefits of breastfeeding
- Milk production
- Breastfeeding positions
- Latching/milk transfer
- Prevention and management of problems
- Recognizing feeding cues

Provide emotional support

Also, the lactation consultant will address the following:

- Teach proper use of breast pump
- Supply mother with pumping supplies
- Discuss safe milk storage practice
- Answer mother’s questions, address concerns
- Discuss follow-up plans

**Follow-up sessions:**

- Same as initial consultation
- Weigh baby
- Refer appropriately if concerns arise regarding baby’s health

**Appendix 4**

**Parent Diary**

**Patient Study Number: _________**

Please use this diary to record the types and amounts of food you have given your baby, the medical attention your baby has required, and the breastfeeding advice and help you have received.

This information should be recorded at the following times:

- one week after discharge from hospital
- when your baby is 2 months old
- when your baby is 4 months old
- when your baby is 6 months old

A research assistant will call you at these times to remind you to complete this diary. She will also ask you the same questions.

**REMEMBER**, please DO NOT disclose to this research assistant which study group you were assigned to (Lactation Consultant or Standard Care).

At the end of the study, please mail this diary back to:

Research Coordinator – Breastfeeding Study

Rm. R240

Children's Hospital of Eastern Ontario

Department of Pediatrics

401 Smyth Road

Ottawa, Ontario

K1H 8L1

If you have questions, please contact:

___________________, Research Coordinator, Breastfeeding Study

(613) 737-7600 x______

____________@cheo.on.ca

**Please complete 1 week post-hospital discharge:** ___________________ (date)

**Please record the types and quantities of food sources your child has been given or is currently receiving (please check and complete all sections that apply):**

□ *Breast Milk*

□ Currently, my child feeds at the breast an average of ____ times per 24 hour period. Each feeding lasts approximately ____ minutes.

□ Currently, my child receives expressed breast milk ____ times per 24 hour period. Each feeding is approximately ____ mL or ____ ounces.

□ My child is exclusively breast fed (receives only breast milk).

□ I completely stopped breastfeeding on _________________ (date)

□ *Formula*

□ I introduced my baby to formula on ________________ (date)

□ Currently, my child receives formula an average of ____ times per 24 hour period. Each feeding is approximately ____ mL or ____ ounces.

□ I stopped all formula on _________________ (date)

□ *Other milk (cow’s milk, soy milk, rice milk, goat’s milk, etc.)*

□ I introduced my baby to other milk on ________________ (date)

□ Currently, my child receives other milk an average of ____ times per 24 hour period. Each feeding is approximately ____ mL or ____ ounces.

□ I stopped all other milk on _________________ (date)

□ *Solids (pabulum, cereals, other prepared foods)*

□ I introduced my baby to solids on ________________ (date)

□ Currently, my child receives solids an average of ____ times per 24 hour period.

□ I stopped all solids on _________________ (date)

**Since discharge from hospital . . .**

**Please record information about any medical assessments your child has had (family doctor or pediatrician, ED, hospital visit or admission):**

Date Provider Reason

________ ______________ ____________________________________________________________

________ ______________ ____________________________________________________________

________ ______________ ____________________________________________________________

**If you have seen your child’s family doctor or pediatrician, please describe any advice or help you have received regarding breastfeeding:**

*Date Description of advice or help*

________ ______________________________________________________________________________

________ ______________________________________________________________________________

________ ______________________________________________________________________________

**If you have sought advice or help regarding breastfeeding from someone else, please describe who you approached (private lactation consultant, public health nurse, public breastfeeding clinic, friend, family member, other) and the advice you received:**

*Source Description of advice or help*

________ ______________________________________________________________________________

________ ______________________________________________________________________________

________ ______________________________________________________________________________

**Other comments or information that you think might be relevant to the study:**

**Please complete when your child is 2 months of age:** __________________ (date)

**Please record the types and quantities of food sources your child has been given or is currently receiving (please check and complete all sections that apply):**

□ *Breast Milk*

□ Currently, my child feeds at the breast an average of ____ times per 24 hour period. Each feeding lasts approximately ____ minutes.

□ Currently, my child receives expressed breast milk ____ times per 24 hour period. Each feeding is approximately ____ mL or ____ ounces.

□ My child is exclusively breast fed (receives only breast milk).

□ I completely stopped breastfeeding on _________________ (date)

□ *Formula*

□ I introduced my baby to formula on ________________ (date)

□ Currently, my child receives formula an average of ____ times per 24 hour period. Each feeding is approximately ____ mL or ____ ounces.

□ I stopped all formula on _________________ (date)

□ *Other milk (cow’s milk, soy milk, rice milk, goat’s milk, etc.)*

□ I introduced my baby to other milk on ________________ (date)

□ Currently, my child receives other milk an average of ____ times per 24 hour period. Each feeding is approximately ____ mL or ____ ounces.

□ I stopped all other milk on _________________ (date)

□ *Solids (pabulum, cereals, other prepared foods)*

□ I introduced my baby to solids on ________________ (date)

□ Currently, my child receives solids an average of ____ times per 24 hour period.

□ I stopped all solids on _________________ (date)

**Since you last recorded in this book . . .**

**Please record information about any medical assessments your child has had (family doctor or pediatrician, ED, hospital visit or admission):**

Date Provider Reason

________ ______________ ____________________________________________________________

________ ______________ ____________________________________________________________

________ ______________ ____________________________________________________________

**If you have seen your child’s family doctor or pediatrician, please describe any advice or help you have received regarding breastfeeding:**

*Date Description of advice or help*

________ ______________________________________________________________________________

________ ______________________________________________________________________________

________ ______________________________________________________________________________

**If you have sought advice or help regarding breastfeeding from someone else, please describe who you approached (private lactation consultant, public health nurse, public breastfeeding clinic, friend, family member, other) and the advice you received:**

*Source Description of advice or help*

________ ______________________________________________________________________________

________ ______________________________________________________________________________

________ ______________________________________________________________________________

**Other comments or information that you think might be relevant to the study:**

**Please complete when your child is 4 months of age:** __________________ (date)

**Please record the types and quantities of food sources your child has been given or is currently receiving (please check and complete all sections that apply):**

□ *Breast Milk*

□ Currently, my child feeds at the breast an average of ____ times per 24 hour period. Each feeding lasts approximately ____ minutes.

□ Currently, my child receives expressed breast milk ____ times per 24 hour period. Each feeding is approximately ____ mL or ____ ounces.

□ My child is exclusively breast fed (receives only breast milk).

□ I completely stopped breastfeeding on _________________ (date)

□ *Formula*

□ I introduced my baby to formula on ________________ (date)

□ Currently, my child receives formula an average of ____ times per 24 hour period. Each feeding is approximately ____ mL or ____ ounces.

□ I stopped all formula on _________________ (date)

□ *Other milk (cow’s milk, soy milk, rice milk, goat’s milk, etc.)*

□ I introduced my baby to other milk on ________________ (date)

□ Currently, my child receives other milk an average of ____ times per 24 hour period. Each feeding is approximately ____ mL or ____ ounces.

□ I stopped all other milk on _________________ (date)

□ *Solids (pabulum, cereals, other prepared foods)*

□ I introduced my baby to solids on ________________ (date)

□ Currently, my child receives solids an average of ____ times per 24 hour period.

□ I stopped all solids on _________________ (date)

**Since you last recorded in this book . . .**

**Please record information about any medical assessments your child has had (family doctor or pediatrician, ED, hospital visit or admission):**

Date Provider Reason

________ ______________ ____________________________________________________________

________ ______________ ____________________________________________________________

________ ______________ ____________________________________________________________

**If you have seen your child’s family doctor or pediatrician, please describe any advice or help you have received regarding breastfeeding:**

*Date Description of advice or help*

________ ______________________________________________________________________________

________ ______________________________________________________________________________

________ ______________________________________________________________________________

**If you have sought advice or help regarding breastfeeding from someone else, please describe who you approached (private lactation consultant, public health nurse, public breastfeeding clinic, friend, family member, other) and the advice you received:**

*Source Description of advice or help*

________ ______________________________________________________________________________

________ ______________________________________________________________________________

________ ______________________________________________________________________________

**Other comments or information that you think might be relevant to the study:**

**Please complete when your child is 6 months of age:** __________________ (date)

**Please record the types and quantities of food sources your child has been given or is currently receiving (please check and complete all sections that apply):**

□ *Breast Milk*

□ Currently, my child feeds at the breast an average of ____ times per 24 hour period. Each feeding lasts approximately ____ minutes.

□ Currently, my child receives expressed breast milk ____ times per 24 hour period. Each feeding is approximately ____ mL or ____ ounces.

□ My child is exclusively breast fed (receives only breast milk).

□ I completely stopped breastfeeding on _________________ (date)

□ *Formula*

□ I introduced my baby to formula on ________________ (date)

□ Currently, my child receives formula an average of ____ times per 24 hour period. Each feeding is approximately ____ mL or ____ ounces.

□ I stopped all formula on _________________ (date)

□ *Other milk (cow’s milk, soy milk, rice milk, goat’s milk, etc.)*

□ I introduced my baby to other milk on ________________ (date)

□ Currently, my child receives other milk an average of ____ times per 24 hour period. Each feeding is approximately ____ mL or ____ ounces.

□ I stopped all other milk on _________________ (date)

□ *Solids (pabulum, cereals, other prepared foods)*

□ I introduced my baby to solids on ________________ (date)

□ Currently, my child receives solids an average of ____ times per 24 hour period.

□ I stopped all solids on _________________ (date)

**Since you last recorded in this book . . .**

**Please record information about any medical assessments your child has had (family doctor or pediatrician, ED, hospital visit or admission):**

Date Provider Reason

________ ______________ ____________________________________________________________

________ ______________ ____________________________________________________________

________ ______________ ____________________________________________________________

**If you have seen your child’s family doctor or pediatrician, please describe any advice or help you have received regarding breastfeeding:**

*Date Description of advice or help*

________ ______________________________________________________________________________

________ ______________________________________________________________________________

________ ______________________________________________________________________________

**If you have sought advice or help regarding breastfeeding from someone else, please describe who you approached (private lactation consultant, public health nurse, public breastfeeding clinic, friend, family member, other) and the advice you received:**

*Source Description of advice or help*

________ ______________________________________________________________________________

________ ______________________________________________________________________________

________ ______________________________________________________________________________

**Other comments or information that you think might be relevant to the study:**

____________________________________________________________________________________________

____________________________________________________________________________________________

__________________________________________________________________

**Appendix 5**

**Nomogram for treatment of hyperbilirubinemia**

**Appendix 6**

**Data collection form**

**Patient Study Number:______________**

Language most comfortable with:

English

French

Mother’s age

Mother’s education level

Elementary school / some high school

Completed high school

Vocational / technical training (post high school)

Some university training

Completed university

Household income

Under 30 000$

From 30 000$ to 70 000$

From 70 000$ to 100 000$

Above 100 000$

Declined to answer

Maternal smoking at home

Yes

No

If yes, how much?

Does anyone else smoke at home?

Yes

No

Mother’s marital status

Married / common-law

Single

How many children prior to patient?

How many breastfed children prior to patient?

Did you seek breastfeeding support with these infants?

Who followed the pregnancy?

Obstetrician

Family physician

Midwife

Other (specify)

No prenatal care

Attendance at prenatal classes?

Yes

No

Will mother go back to work before child is one?

Yes

No

If yes, when?

Baby’s age at admission to CHEO?

Baby’s birth weight?

Gestational age at birth?

Baby’s weight on admission to CHEO?

Baby’s weight on discharge from CHEO?

Urine culture done?

Yes

No

Blood culture done?

Yes

No

Cerebrospinal fluid culture done?

Yes

No

Antibiotic therapy given?

Yes

No

Intravenous fluids given?

Yes

No

Duration & volume of fluid given before weaned off

Duration of phototherapy in hours

Length of hospital stay in days

Need for formula supplementation while in hospital?

Yes

No

Formula given prior to hospitalization?

Yes

No

**Appendix 7**

**Qualitative Interview for the Intervention Group**

**After the intervention:**

We want to understand your experiences working with the CHEO lactation consultant during your hospital stay and during the follow-up sessions. Please describe how you felt about breast feeding prior to meeting with the lactation consultant, and any concerns or specific questions about breastfeeding that you hoped to have answered.

Please describe how you felt during the meeting. Was the encounter a positive experience?

Please explain which aspects of the first meeting in hospital you found helpful, versus those that you felt were not helpful. Was there one component that you felt was most helpful?

Please describe how you felt after the first session. Were your concerns and questioned addressed?

Please explain which aspects of the follow-up sessions you found helpful, versus those that you felt were not helpful. Was there one component that you felt was most helpful?

Please share your thoughts on the number and frequency of the follow-up sessions.

Please describe how you felt when your child was admitted, and when he/she was discharged from the hospital.

Please describe how confident you felt with your breastfeeding skills and what else could have been done at CHEO to help you.

If this intervention were to be used as a model for a breastfeeding support program at CHEO for all mothers, what recommendations would you have?

**At the end of the study period:**

We want to understand your experiences working with the CHEO lactation consultant during your hospital stay and during the follow-up sessions.

When we spoke last, you seemed to feel that X was the most useful component of the intervention. Now that some time has passed, is this still how you feel?

Now that some time has passed, do you have other recommendations to make, should this intervention be used as a model for a breastfeeding support program at CHEO for all mothers?

If you needed additional help with breastfeeding after the meetings with the lactation consultant were over, please describe how you addressed your need.

If you discontinued breastfeeding after your child’s admission to the hospital, please describe if anything could have been done differently at CHEO to help you continue.

**Appendix 8**

**Qualitative Interview for the Control Group**

**One week post hospital discharge:**

We want to understand your experiences while your child was hospitalized at CHEO.

Please describe how you felt when your child was admitted, and when he/she was discharged from the hospital.

Please describe how confident you felt with your breastfeeding skills.

If you had questions/concerns regarding breastfeeding, please describe how these were addressed.

Please describe whether you felt you received adequate breastfeeding support and help during your stay.

Please describe what could have been done to improve your experience at CHEO in terms of breastfeeding support.

**At the end of the study period:**

We want to understand your experiences after your child was discharged from CHEO.

When we spoke last, you seemed to feel “a certain way X” about your breastfeeding skills. Now that some time has passed, is this still how you feel?

Do you feel your breastfeeding needs were addressed adequately when you were at CHEO? What else could have been done to help you.

If you needed additional help with breastfeeding after you were discharged from the hospital, please describe how you addressed your need.

If you discontinued breastfeeding after your child’s admission to the hospital, please describe if anything could have been done differently at CHEO to help you continue.

**Appendix 9**

**Sensitivity analysis based on different breastfeeding rates at baseline and expected effect size**

| **Breastfeeding rate at baseline** | **Effect** | **Sample Size** |
| --- | --- | --- |
| 30% | 15% | 163 |
| 30% | 25% | 61 |
| 30% | 35% | 31 |
| 40% | 15% | 173 |
| 40% | 25% | 62 |
| 40% | 35% | 31 |
| 50% | 15% | 170 |
| **50%** | **25%** | **58** |
| 50% | 35% | 27 |
